# Pilot postal birth cohort hepatitis C virus screening in UK primary care: HepCAPP study

**DOI:** 10.3399/BJGP.2024.0219

**Published:** 2025-01-28

**Authors:** Ruth Simmons, Annabel A Powell, Samreen Ijaz, Sema Mandal, Justin Shute, Yasmin Mohammadi, Michael Lattimore, Kelsey McOwat, Hannah L Moore, Aisling O’Rourke, Monica Desai, John Macleod, Asra Asgharzadeh, Zoe Ward, Peter Vickerman, Ross J Harris, Graham R Foster, Kirsty Roberts, Matthew Hickman

**Affiliations:** UK Health Security Agency, London, UK.; UK Health Security Agency, London, UK.; UK Health Security Agency, London, UK.; UK Health Security Agency, London, UK.; UK Health Security Agency, London, UK.; UK Health Security Agency, London, UK.; UK Health Security Agency, London, UK.; UK Health Security Agency, London, UK.; UK Health Security Agency, London, UK.; Population Health Sciences, Bristol Medical School, University of Bristol, Bristol, UK.; UK Health Security Agency, London, UK.; Centre for Academic Primary Care, Population Health Sciences, University of Bristol, Bristol.; Population Health Sciences, Bristol Medical School, University of Bristol, Bristol and NIHR Health Protection Research Unit in Evaluation of Interventions, University of Bristol, Bristol.; Population Health Sciences, Bristol Medical School, University of Bristol, Bristol and NIHR Health Protection Research Unit in Evaluation of Interventions, University of Bristol, Bristol.; Population Health Sciences, Bristol Medical School, University of Bristol, Bristol and NIHR Health Protection Research Unit in Evaluation of Interventions, University of Bristol, Bristol.; UK Health Security Agency, London, UK.; Centre for Immunobiology, Queen Mary University of London, London, UK.; Population Health Sciences, Bristol Medical School, University of Bristol, Bristol, UK.; Population Health Sciences, Bristol Medical School, University of Bristol, Bristol and NIHR Health Protection Research Unit in Evaluation of Interventions, University of Bristol, Bristol.

**Keywords:** birth cohort screening, cost per case found, hepatitis C, primary health care

## Abstract

**Background:**

Birth cohort screening has been implemented in some countries to identify the potentially ‘missed population’ of people with undiagnosed chronic hepatitis C virus (HCV) who may not be found through targeted approaches.

**Aim:**

To determine uptake of HCV antibody testing using an oral swab screening method, the overall yield, whether those testing positive had risk markers in their primary care record, and the cost per case detected.

**Design and setting:**

This was a pilot screening study set in general practices in the Southwest of England, Yorkshire and Humber, and South London.

**Method:**

Participants consenting were sent an oral swab kit in the post and saliva samples were tested for antibodies to HCV.

**Results:**

In total, 16 436/98 396 (16.7%) patients consented and were sent an oral swab kit. Of these, 12 216 (12.4%) returned a kit, with 31 participants (yield 0.03%) testing positive for HCV antibodies. Of those positive, 14/35 (45%) had a risk marker for HCV on their primary care record. Two (yield 0.002%) were confirmed RNA positive and referred for treatment, both had HCV risk markers. The cost per case was £16 000 per HCV antibody detected and £247 997 per chronic HCV detected.

**Conclusion:**

Wide-scale screening could be delivered and identify people infected with HCV, however, most of these individuals could have been detected through lower-cost targeted screening. The yield and cost per case found in patients were substantially worse than model estimates and targeted screening studies. Birth cohort screening should not be rolled out in primary care in England.

## Introduction

Since 2015, widespread roll out of highly effective direct acting antivirals, increased efforts to diagnose, treat, and cure individuals with chronic hepatitis C virus (HCV) infection has reduced chronic HCV in England by 51.6% to 62 600.^1^ Targeted HCV screening in primary care, drug treatment, and other settings are cost-effective.^2^^,^^3^

In England over 85% of HCV transmission and the greatest burden of HCV is among people who inject or have injected drugs. It is estimated that between one in three to half of these HCV infections are among people who have ceased injecting, with an unknown proportion among people who may no longer be in contact with drug treatment services and lack any clinical history that could be used to target them for HCV testing and linkage to care *—* such as through primary care risk algorithms.^4^^,^^5^ In part to reach this ‘missed’ population, the USA and some other countries have proposed and implemented birth cohort screening (for example of individuals born 1945–1965).^6^^–^9 In England, evidence for how to reach the ‘unknown/difficult to find’ population is limited. Williams *et al*, hypothesised that adding HCV screening to the NHS health check offered in primary care for people aged 45–70 years could be cost-effective.^10^ However, there is no empirical evidence yet to test this hypothesis and it is unclear whether it could be implemented or have a wide enough population reach.

For the current study, HepCAPP (Hepatitis C Virus CAse Finding in Primary Care Pilot) an alternative model was piloted and adapted, used previously to establish chlamydia prevalence,^11^ where individuals aged between 40 and 64 years in participating general practices were actively invited to have an HCV antibody test using an oral swab that was posted to the individuals’ homes. The overall uptake of the intervention is reported along with HCV antibody positivity in an unselected population, the study also assessed whether those identified as positive could have been identified using a risk-based algorithm and the cost per case found was evaluated.

## Method

### Study design and participants

The study was pre-registered.^12^ HepCAPP was a pilot cohort screening study carried out in general practices in the Southwest of England, Yorkshire and Humber, and South London.^12^ Recruitment of general practices was extended in Leeds to the entirety of the Yorkshire and Humber region to maximise uptake of testing. Individuals aged 40–64 years of age were eligible, registered at a participating general practice, and identified using a search of the GP system. Individuals were excluded if they were receiving palliative care, receiving active cancer treatments, were considered unable to consent (for example, they had diagnosed dementia, learning difficulties, and so on), successfully treated for HCV infection, and/or had selected national data opt out. Patient lists including name, date of birth, NHS number, and address were sent by secure data transfer to the UK Health Security Agency (UKHSA).

**Table:** How this fits in

Targeted hepatitis C virus (HCV) screening in primary care and other settings is highly cost-effective. The NHS England HCV elimination programme has recently shifted to other screening models to identify people who may not be detected through targeted screening. HepCAPP (Hepatitis C Virus CAse Finding in Primary Care Pilot) is the first study in the UK to pilot a birth cohort model based in primary care inviting all patients aged 40–64 years to have an HCV antibody test using an oral swab kit posted to their homes. Acceptability of testing using this method was above NHS England targets, however, the yield was too low and costs too high (compared with theoretical estimates and targeted screening) to recommend implementation.

### Intervention

Between February 2022 and December 2022, eligible individuals were sent an invitation letter by the UKHSA with an electronic link and QR code directing them to the patient information leaflet (PIL) and further details about the study. Individuals were asked to complete an electronic consent form if they wanted to take part. A paper version of both the PIL and the informed consent form were available on request. As part of the consent process individuals were asked to enter demographic information (sex, country of birth, and ethnicity), confirm their contact details, and consent to the collection and analysis of their oral fluid sample for HCV antibody testing. Individuals were also asked to consent to the long-term storage of their oral fluid sample after the close of the study to support further research, but refusal did not have an impact on their inclusion in the study. Individuals could withdraw from the study at any time, and any associated unprocessed oral fluid samples destroyed, and their data removed from the study database. Personal identifiable information on individuals who did not respond or give consent were deleted 3 months after the invite letter was sent. Participation was defined as those who completed the survey and consented to the study.

General practices were asked to run a risk-based algorithm known as the MSD Patient Search Identification (PSI) Tool^13^ on all their patients (similar to an earlier trial: HepCATT^14^) to identify clinical indicators of HCV risk. This was linked to the study participants list who consented to be contacted by UKHSA using NHS numbers, to compare yield of patients with HCV antibodies identified through the study with those having clinical indicators of HCV risk identified by the MSD PSI tool (flagging individuals in one or more of the following groups:

- ‘At risk’,
- ‘In at-risk group and Hep C positive ever’,
- ‘In at-risk group and not Hep C positive’,
- ‘At risk not including HIV screening-related codes’, and
- ‘All patients who have ever been Hep C positive’).

The output was sent by secure data transfer to the UKHSA study team.

Participants were sent an oral fluid home testing kit to their home address that was accompanied by a paper copy of the PIL and a self-sampling instruction leaflet. (Supplementary Information S1 gives detailed descriptions of collection, processing, and analysis of saliva samples, all of which was carried out by UKHSA laboratories).

Participants whose sample tested negative for antibodies to HCV were notified directly by UKHSA by email, or by post if preferred. If a sample was repeatedly positive for antibodies to HCV, UKHSA notified the participant’s GP with a request for the GP to follow-up the individual and to obtain a blood sample for anti-HCV and HCV Ribonucleic acid (RNA) testing. The relevant operational delivery network (ODN) (NHS England structure through which HCV treatment is delivered in England) HCV nurse was also notified by UKHSA of the participant’s details and of the positive HCV antibody result with a request to engage and follow-up with the participant and their GP including referral into an HCV care pathway if diagnosed with chronic HCV disease (that is, HCV RNA positive). A review of the GP and ODN’s actions was conducted by UKHSA for the participants who were anti-HCV positive.

### Outcomes

The primary outcome was uptake of anti-HCV oral fluid testing, defined as the number and percentage of individuals that participated and were sent an oral fluid home testing kit. As HepCAPP was a pilot study, there was no formal sample size calculation. NHS England set the criteria in terms of their expectation that they wanted to see 10 000 people tested and at least 10% return from inviting 100 000 people. This would give NHS England sufficient information to consider whether it was worth implementing across England. It was also sufficient power to test whether the yield of people with chronic HCV cases was substantially below 0.2%. Secondary outcomes were yield in terms of:

- number and percentage of participants whose samples had detectable HCV antibodies; and
- number and percentage of participants assessed for and diagnosed with chronic HCV;
- number and percentage of participants with HCV antibodies that would be flagged and targeted for HCV testing using the MSD PSI tool; and
- cost per case detected and potential cost-effectiveness.

### Costs data

Information on costs of HCV testing by this approach were also collected in a spreadsheet template in terms of practice time involved in running the initial search and MSD PSI tool, and UKHSA/University of Bristol’s time in organising and administering patient invitations, managing and following-up requests for oral fluid home testing kits, anti-HCV test processing, and coordinating results to general practices, participants, and ODNs. The overall spending is presented in two main categories: variable costs and fixed costs.

Variable costs incorporate a range of elements, including costs related to kits, delivery services, postage, paper PILs, and the consent process. In contrast, fixed costs specifically encompass staffing expenses. These costs can estimate average cost per practice, patient tested, and patient with HCV antibodies detected. Cost figures were determined in British pounds as of the year 2022. A decision on whether to adapt and update the earlier cost-effectiveness model^10^ depended on whether there was a sufficient number of people with chronic HCV detected (and who could not be identified through targeted case finding).

### Statistical analysis

This is a pilot study so no formal sample calculation was undertaken apart from prior expectation from NHS England. All summary statistics for data are presented as absolute values with percentages, where data reported (that is, with missing excluded). Multivariable logistic regression was used to assess the association between consenting and certain characteristics including, where available, sex, age, Indices of Multiple Deprivation (IMD) score, ethnicity, and country of birth. In regression analysis, odds ratios (ORs) are presented with 95% confidence intervals (CIs). Associations between explanatory variables and returning a sample were tested for statistical significance at *P*<0.05 using the Chi-squared test. Data were managed in Microsoft SQL Server Management Studio and results were analysed in Stata (Release 17).

## Results

In total, 98 396 individuals aged 40–64 years and registered at 25 participating general practices across the three regions in England, the Southwest, Yorkshire and the Humber, and South London, were invited to take part in the HepCAPP study. A reduced number of general practices were included because of the study reaching the NHS England pre-defined success criterion.^12^ Overall, 16 436 (16.7%) participants consented to being part of the study and were sent oral swab test kits, and 12 216 (12.4%) returned a sample to UKHSA. The approach to HCV antibody screening was, overall, accepted well by registered patients as there were minimal complaints and general queries related mainly to requests for a paper consent form and PIL, invalid results, and requests to opt out of the study.

**Figure 1.**
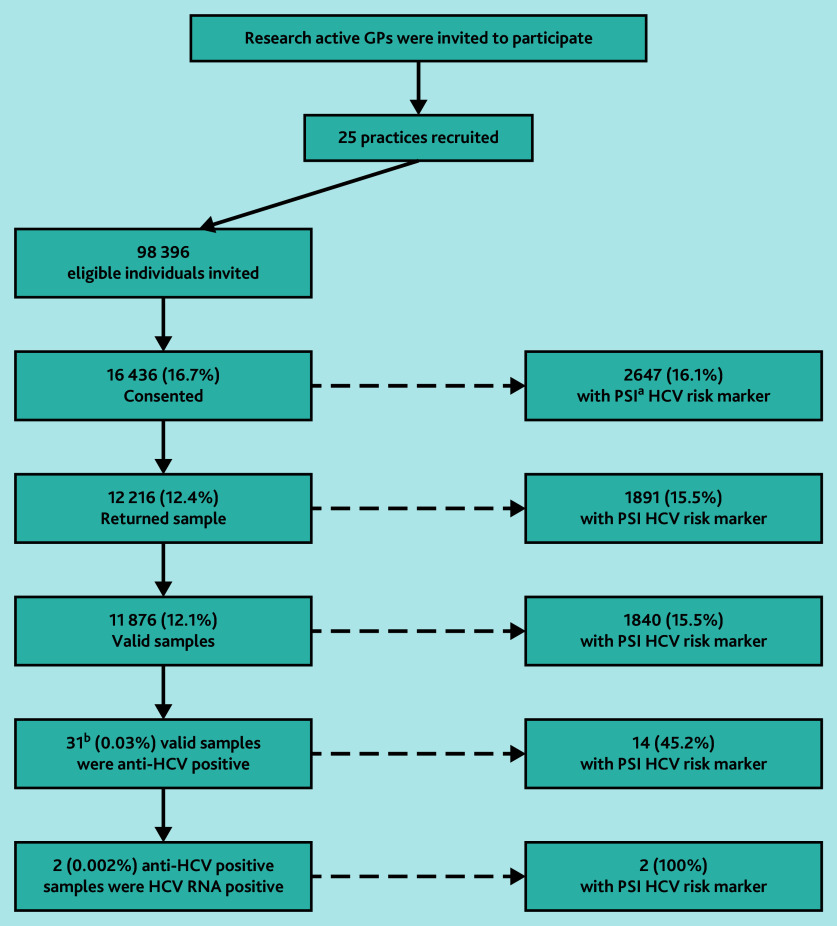
Flow diagram. ^a^‘PSI’ refers to the Patient Search Identification tool — a case-finding tool that searches for coded hepatitis C virus risk factors in patient records in primary care settings. The PSI tool was provided and funded by MSD (UK) Ltd as part of the services tendered to NHS England to support the NHS England Hepatitis C Elimination Strategy. ^b^In total 11/31 subsequently confirmed anti-HCV by blood test. HCV = hepatitis C virus.

Of those consenting and those returning a sample, 2647 (16.1%) and 1891 (15.5%), respectively, had HCV risk markers identified using the PSI tool. There were 11 876 valid samples from individuals (12.1%); of these 31/11 876 (0.03% yield of those invited) had HCV antibodies detected, that is, were likely to have been ever infected; of which 14 (45.2%) were in people with PSI HCV risk markers (Figure 1) . After follow-up with GPs for confirmatory testing, 28 individuals had a follow-up blood sample taken to test for HCV antibodies of which 11 were confirmed positive. All these individuals had an HCV RNA test, two (0.002% yield of those invited) were found to be positive and are being treated. Both individuals had risk factors identified through the PSI tool.

Median age of those invited was 52 years (interquartile range 46–58 years), 61.1% (60 139) were registered at a general practice in the Southwest, 20.4% (20 077) in Yorkshire and the Humber, and 18.5% (18 180) in South London (Table 1).

**Table 1.** Characteristics of individuals invited to the study and by those consenting to the study, returning a kit and a valid sample^a^

**Characteristic**	**Overall**		**Consented**				**Returned kit**				**Valid sample**			

	**Invited**		**Overall**		**In PSI^b,c^**		**Overall**		**In PSI**		**Overall**		**In PSI**	

	***n***	**%**	***n***	**%**	***n***	**%**	***n***	**%**	***n***	**%**	***n***	**%**	***n***	**%**
**Total**	98 396		16 436		2647		12 216		1891		11 876		1840	

**Sex**														
Female	–	–	9109	55.4	1720	65.0	6759	55.3	1225	64.8	6639	55.9	1199	65.2
Male	–	–	7308	44.5	921	34.8	5445	44.6	665	35.2	5226	44.0	640	34.8
Missing	–	–	19	0.1	6	0.2	12	0.1	1	0.1	11	0.1	1	0.1

**Age group, years^d^**														
40–44	20 527	20.9	2672	16.3	705	26.6	1761	14.4	462	24.4	1717	14.5	456	24.8
45–49	19 203	19.5	2753	16.7	612	23.1	1964	16.1	431	22.8	1898	16.0	418	22.7
50–54	20 344	20.7	3214	19.6	548	20.7	2310	18.9	388	20.5	2229	18.8	368	20.0
55–59	20 381	20.7	3839	23.4	450	17.0	2929	24.0	342	18.1	2840	23.9	335	18.2
60–64	17 343	17.6	3958	24.1	332	12.5	3251	26.6	268	14.2	3191	26.9	263	14.3
Missing	589	0.6					1	0.0						

**Region**														
South West	60 139	61.1	11 352	69.1	1440	54.4	8444	69.1	1007	53.3	8205	69.1	979	53.2
Yorkshire and	20 077	20.4	3052	18.6	258	9.7	2270	18.6	187	9.9	2196	18.5	178	9.7
Humber														
South London	18 180	18.5	2032	12.4	949	35.9	1502	12.3	697	36.9	1475	12.4	683	37.1

**Ethnicity**														
Asian	–	–	238	1.4	68	2.6	172	1.4	45	2.4	166	1.4	45	2.4
Black	–	–	56	0.3	32	1.2	37	0.3	25	1.3	37	0.3	25	1.4
White	–	–	15 545	94.6	2357	89.0	11 618	95.1	1691	89.4	11 298	95.1	1644	89.3
Mixed	–	–	124	0.8	47	1.8	73	0.6	30	1.6	72	0.6	29	1.6
Other	–	–	48	0.3	22	0.8	28	0.2	15	0.8	28	0.2	15	0.8
Missing	–	–	425	2.6	121	4.6	288	2.4	85	4.5	275	2.3	82	4.5

**IMD quintile**														
1 (most deprived)	14 608	14.8	1639	10.0	341	12.9	1155	9.5	239	12.6	1112	9.4	231	12.6
2	17 728	18.0	2638	16.1	410	15.5	1889	15.5	286	15.1	1829	15.4	277	15.1
3	20 202	20.5	3198	19.5	508	19.2	2336	19.1	350	18.5	2270	19.1	340	18.5
4	20 085	20.4	3713	22.6	595	22.5	2783	22.8	414	21.9	2701	22.7	407	22.1
5 (least deprived)	25 702	26.1	5242	31.9	792	29.9	4047	33.1	601	31.8	3958	33.3	584	31.7
Missing	71	0.1	6	0.0	1	0.0	6	0.0	1	0.1	6	0.1	1	0.1

a *Characteristics also shown for those identified as in a risk group for each study outcome.*

b *‘PSI’ refers to the Patient Search Identification tool — a case-funding tool that searches for coded hepatitis C virus risk factors in patient records in primary care settings.*

c *Numbers overall who appeared in the PSI search are non-contemporaneous with lists extracted to be invited to the study. Patient lists for invitations were extracted between January and November 2022, and PSI searches were extracted between January and May 2023.*

d *Values have been rounded. IMD = Index of Multiple Deprivation.*

Of the 16 436 consenting individuals, 44.5% (7308) were male and 94.6% (15 545/16 436) were of White ethnic origin. A higher proportion of individuals consenting (31.9%, *n* = 5242) were resident in the least deprived quintile, compared with the most deprived quintile (10.0%, *n* = 1639). In multivariable logistic regression analyses, likelihood of participation increased with age and decreasing levels of deprivation, and with individuals in London least likely to participate compared with the Southwest and Yorkshire and Humber (Table 2).

**Table 2.** Multivariable logistic regression to examine factors associated with consenting to the HepCAPP (NHS England Hepatitis C Virus CAse Finding in Primary Care Pilot) study

**Factor**	**Adjusted ORs**	**95% CI**	***P*>*z***
**Age**	1.03	1.027 to 1.032	<0.001

**IMD quintile**			
1 (most deprived)	1		
2	1.32	1.23 to 1.41	<0.001
3	1.46	1.37 to 1.55	<0.001
4	1.80	1.69 to 1.92	<0.001
5 (least deprived)	2.12	1.99 to 2.25	<0.001

**Region**			
South West	1.85	1.76 to 1.94	<0.001
Yorkshire and Humber	1.30	1.22 to 1.38	<0.001
South London	1		

*CI = confidence interval. IMD = Index of Multiple Deprivation. OR = odds ratio.*

For the 14 people with HCV antibodies and a PSI HCV risk marker recorded, six had been previously recorded as positive for HCV and eight were recorded as having a risk factor but not known to be HCV positive.

### Cost per case detected

The fixed costs, totalling £350 180, were designated for staff expenses with variable costs amounting to £145 814 and total cost in 2022 reached £495 994. The unit cost per patient was £28.70; the unit cost per HCV antibody-positive test was £16 000 per case identified (£11 296 in fixed and £4704 in variable costs); and the unit cost for each chronic HCV case detected was £247 997 (Supplementary Table S1).

Given the low yield of chronic HCV cases and the considerably higher detection costs than assumed from birth cohort models in the UK^10^ the authors of the current study decided any further economic evaluation or calculating the incremental cost-effectiveness ratio would not be worthwhile or informative.

## Discussion

### Summary

The acceptability and uptake of wide-scale HCV screening using the approach described in HepCAPP were above expectation at 12.4% (12 216) of nearly 100 000 individuals. However, the yield was very low with 0.03% (*n* = 31) identified as positive and two people (0.002%) with chronic HCV infection. Both the people with chronic HCV had a PSI HCV risk flag and could have been detected through more targeted screening, which is being rolled out across England. Uptake of testing also tended to be lower in younger people and more disadvantaged communities. The cost per case was £16 000 per HCV antibody detected and £247 997 per chronic HCV detected.

### Strengths and limitations

A key strength of this study was that this was the first pilot study to be conducted in the UK of wide-scale HCV screening of the general public where nearly 100 000 individuals were invited for opt-in testing to be carried out at their homes. However, it is acknowledged that there were also several limitations to the current study. First, the use of oral fluid testing meant that those identified with an antibody-positive test had to have additional tests for their RNA status. This adds additional steps in the care pathway and risk of a person being lost to follow-up. The oral fluid testing also resulted in a high number of invalid tests, 304/12 216 (2.8%) of individuals never returned a valid sample despite including detailed instructions and a QR link to a video on how to take the test. As a result of the limitations of the oral fluid anti-HCV test, there was also a high percentage of samples that subsequently tested negative in their follow-up blood sample test for HCV antibodies (20/31, 64.5%). An evaluation determining the sensitivity and specificity of the HCV antibody assay has previously indicated the assay to have a sensitivity of 92% and specificity of 99%.^15^ The low-levels signals seen in this study did all cluster towards the bottom end of the assay around the cut-off. This may reflect the performance of the assay in a large, unselected population. The cut-off of the assay could have been moved to reflect this, however, it provided a better understanding of the low-level reactives in particular in the context of declining HCV antibody levels following viral clearance. In the context of the number of samples that were tested, the false-positive rate is low. With respect to false negatives, it was anticipated that those individuals who were HCV-infected and HCV RNA positive would have a high antibody level, similar to what is seen in serum samples. Therefore, even with the lower sensitivity levels of the oral fluid assay, it was unlikely that samples from those individuals who were HCV RNA positive would be missed.

Second, the general practices taking part were currently research active and received some compensation. Therefore, additional incentives may be required if this model of testing across UK primary care practices was rolled out. The geographical location and number of general practices were updated from what was stated in the published protocol in response to both the uptake of general practices taking part and uptake of anti-HCV testing. Third, although HepCAPP was feasible, uptake was higher in those of White ethnic origin and higher in the least deprived quintiles. Further interventions and investment would be required to access the underrepresented groups.

### Comparison with existing literature

Compared with other birth cohort screening studies, the anti-HCV was very low (0.03% versus 3.25% in the USA,^6^ 0.36% in Japan,^7^ and 3.2% in Canada^16^). The low yield also contributed to the HepCAPP yield being 25 to 50 times more costly than with other studies.^10^ A recent trial of targeted screening estimated a cost of £1305 per HCV antibody and £5569 per chronic HCV tested.^14^ The theoretical model of birth cohort screening in UK primary care estimated a substantially lower yield than observed in the current study in practice.^10^ However, the form of HCV birth cohort screening in the current study was based on a general invitation rather than being incorporated into NHS health checks (as this was not possible to pilot at the time, and NHS health checks although achieving approximately 40% uptake were not running at all practices.^17^). It is possible that there would be a higher proportion of people with chronic HCV (and no risk markers) who did not respond to the current study’s invitation but who would take up NHS health checks and could be detected, but the proportion would have to be several times higher than was observed in HepCAPP to be cost-effective.

The current study raises the hypothesis that there are fewer undiagnosed people than expected who need non-selective case-finding approaches, however, other evidence (from screening of unselected patients) is needed to corroborate this hypothesis or prompt alternative birth cohort designs.

In the UK, further pilots are underway to test other screening models for people who are not aware that they are at risk, including ones in primary care, through web testing, and in antenatal services.^18^ One of the major programmes is the introduction of opt-out blood-borne virus testing in emergency departments across areas of very high diagnosed HIV prevalence across England. The 12-month evaluation report for the emergency department opt-out programme indicates that new HCV RNA diagnoses was higher among males, people aged ≥35 years, people of White other ethnicity, with little difference across IMD quintiles.^19^

### Implications for practice

The yield from HepCAPP suggests that the approach used for birth cohort screening in primary care in the current study is too low to warrant its national roll out. Instead in primary care targeted screening that identifies people for HCV testing who have had an injecting history or other risk markers *—* as shown by HepCATT^14^ and the MSD PSI algorithm in place in primary care systems *—* is highly cost-effective and should continue to be implemented. The intervention does involve some financial support for practices to run the targeted screening, although some further implementation may be required so that practices can implement it automatically and reduce the number of people flagged and the steps in the process.^20^ Additionally, the NHS England HCV elimination programme has invested in other pathways to find people with HCV who may not be detected through targeted screening in primary care or other sites (such as drug treatment centres or prisons^3^^,^^21^). These include opt-out testing in selected emergency rooms in England and an NHS England web-based online testing portal.

## Data

Data is available upon request from corresponding author.
